# The Annual Reminder of Hospital Sunday

**Published:** 1918-06-22

**Authors:** 


### Hospital Sunday, June 23, 1918.

### SPECIAL HOSPITAL SUNDAY FUND SECTION.

### HOW TO REVIVE CHURCH LIFE.

### The Annual Reminder of Hospital Sunday.

In these times of numerous Flag Days and special collections in the churches for various objects more or less extraneous to the immediate interests of the churches, there is a danger lest the claim of Hospital Sunday may be made perfunctorily from many pulpits. It is well, therefore, to remind the clergy, as well as their congregations, that Hospital Sunday was the first of Flag Days, and that it was not sprung upon them from without, but the spontaneous creation of one of themselves, the late Canon Miller, of Birmingham. The traditional relation of the Church to the sick from the time when monastic infirmaries were the first public hospitals fully accounted for Canon Miller's action, which, however, had a larger hope to fulfil. He knew, as the clergy must know, that there are many people who fail to make religion part of their daily lives, and he therefore preached to them the week-day gospel of personal service, as a thank-offering on the part of the healthy, a free gift of themselves in the cause of the sick, among whom chance or disease at any moment might include them. The text "Do my commandments and ye shall know of the doctrine" is an old one, and those in doubt and hesitancy of faith can do no better towards resolving their difficulties than to undertake the nearest duty which lies to hand. Since, however, all cannot become active workers for the voluntary hospitals on committees or Linen Guilds, or the like, the chance to give on Hospital Sunday is open to all. This gospel of personal service, which is the root of all the sermons preached on Hospital Sunday, is, as it were, the Laodicean's religious opportunity, for its appeal to generous instinct and the opportunity which it provides for active work on behalf of the helpless, is so direct that even the sceptic responds to it. Had the Churches realised the opportunity which Canon Miller gave them to bring home this, the simplest lesson of Christianity, their congregations would be more active co-operators with their ministers than they are to-day.

The war has taught us all, even the extremest individualist (the conscientious objector), that we are all members one of another, and that no man can stand aside and say that he will take neither part nor lot in the fate of his country. What is this but the recognition of the duty of personal service in its crudest form? Should it be so hard, then, to show that if we must fight against our human enemies without, and stand by each other in that contest, we are equally bound personally to serve against the enemies of disease, and personally to aid those who suffer from its onslaught? The latter, too, appeals to the highest in ourselves, for it is constructive work, in which the heart will not sicken as even Captain Ball said his did when one German airman after another crashed to earth a victim to his skill. All this, the glory of saving life, is the glory of the hospitals, and in time of war we can turn from the carnage, which evil passions have created, to this the proper fighting-ground of man. The hospitals keep the divine flag flying, and for this service alone they deserve and receive unfailing support.

Were the meaning of personal service brought home to the congregations the clergy would find that it would react beneficially on the whole of Church life. But if the clergy are deaf to its meaning, how should their congregations carry it away? Canon Miller, in founding Hospital Sunday, did more than make a present to the hospitals. He provided the clergy with so direct an example of the claims of personal service that none could deny it, in order that the gospel of personal service itself might be preached with added conviction. Duties are only the applications of general principles to particular cases, and the duty of personal service to the sick is only the most forcible reminder of the principle that we are here "not to be ministered unto, but to minister." Our duty to the sick is so plain, the appeal of pain is so instant, that on Hospital Sunday the clergy have an illustration of a principle of the neglect of which they often complain in Church life.

The want of support for "church expenses," for the maintenance of the fabric of the building, and for the conduct of the essential activities of parochial life, has led some clergy to make deductions from special collections. This practice vas discussed and condemned, for obvious reasons, in The Hospital a year ago. Our present point is merely this, that if the clergy had really preached the gospel of personal service, of which Hospital Sunday is an annual witness and reminder, it is incredible that the need for such deductions should have arisen. A congregation has a personal duty to its parish church, and a personal responsibility for its proper maintenance. If its sense of *this* is lost, it must be dead indeed. This is one reason why Hospital Sunday should be an annual revival of the corporate spirit, which should be welcomed by the clergy because it provides them with the illustration of a duty on the recognition of which the vigour of their own parish life depends.

